# Clinical outcome and comparison of burn injury scoring systems in burn patient in Indonesia

**DOI:** 10.1016/j.afjem.2021.04.005

**Published:** 2021-06-05

**Authors:** Risa Herlianita, Edi Purwanto, Indri Wahyuningsih, Indah Dwi Pratiwi

**Affiliations:** Department of Nursing, School of Nursing, University of Muhammadiyah Malang, Indonesia

**Keywords:** Burn Injury, Clinical Outcome, Burn prognosis, Injury Scoring System

## Abstract

**Introduction:**

The purpose of this study was to explore and compare the performance of four burn injury scoring systems in Indonesia. In a retrospective study, data of all burn patients admitted to the emergency centre (EC) were collected. The following clinical outcome and four burn injury scoring systems were used to assess each patient: Abbreviated Burn Severity Index (ABSI), Belgian Outcome in Burn Injury (BOBI), the Ryan model, and revised Baux Score.

**Methods:**

From April 2017 to April 2018, clinical outcome and burn injury score for every admitted patient were calculated to evaluate burn prognosis. Demographic information, ABSI score, full-thickness total body surface area (TBSA), overall TBSA, hospital stay, and inhalation injury were noted for analysis. Discriminative ability and goodness-of-fit of the prediction models were determined by receiver operating characteristic curve analysis and Hosmer–Lemeshow tests.

**Results:**

We included 72 patients (mean age: 40.79 ± 16.30 years, average TBSA: 23.59% ± 24.84). Only 1 (1.4%) of them was diagnosed with inhalation injury. Mortality rate was 25%. Deceased patients had significantly higher mean age, %TBSA, and number of inhalation injuries. The ABSI model with sensitivity was 81.6, specificity was 92.5, accuracy was 87.3 and under the Receiver Operator Characteristics curve (AUC) was 0.93 (SE = 0.03).

**Conclusions:**

The best estimation of predicted mortality was obtained with the ABSI model.

## African relevance

• This study could be utilised in an underdeveloped setting, such as Africa.
• The findings of this study might also be compared with other African settings where similar measures were implemented.
• The predictions in this sample supported the excellence ratings, which is another model that can be used across African countries.

## Introduction

Burn injuries are a global public health problem, responsible for an estimated 250,000 deaths per year [1]. As a result, burn injury is one of the world's health issues contributing to the burden of disease and reported at about 180,000 deaths per year, with the highest incidence reported in Southeast Asia, including Indonesia [1]. The major burden of burn injuries comes from low- and middle-income countries (LMICs), accounting for >90% of the global injury burden. According to the World Health Organization (WHO), the three most vulnerable regions are the African region, South East Asian region, and Eastern Mediterranean region, with more than half of the total burden on the South East Asian region [2]. In addition, the WHO predicts that the large majority of post-burn deaths happened in LMICs [1].

Considering this problem, a management strategy predicting the prognosis of the condition of burn patients is needed. Studies have indicated a strong link between burn size and mortality [[3], 4., [5], [6]]. Furthermore, burns that affect at least 20% of the total body surface area are linked to an increased risk of death [6]. As a result, a model that reliably predicts burn mortality may be helpful in assessing clinical path, exploring treatment options with patients and their families, and evaluating new or innovative interventions. Besides evaluating the likelihood of burn patient mortality, accurate and robust prediction models as a standardised method will calculate the efficiency of the burning service [7]. Many prognostic scoring systems have been devised to predict mortality risk in burn patients [3]. It has existed since the mid-20th century [1,5]. For example, Abbreviated Burn Severity Index (ABSI) [8] is considered as one of the easy scoring systems [7]. Another model is Ryan, which is utilised for age, TBSA and inhalational injury [9], and has accounted for the presence of pneumonia and trauma at the time of injury [9]. In addition, the Belgian Outcome of Burn Injury (BOBI) [10] and The FLAMES use a hybrid scoring model, utilising both burn specific risk factors and initial APACHE II scores [11,12].

However, several scoring systems of burn injuries in developing countries have been studied to date with different results. Previous study conducted by Halgas et al. [13] suggested that the revised Baux score was both accurate and easy to calculate, making it clinically useful. In addition, the revised Baux score was the most accurate burn mortality risk score to predict burn mortality in a Malaysian population [14]. In Indonesia, the majority of burn scoring systems used Rayan, Revised Baux Score, BOBI, ABSI. However, all those scoring systems were less validated, and few studies explored the most accuracy burn scoring system in Indonesia. The aim of this study was to identify clinical outcomes and compare the performance of four burn injury scoring systems in Indonesia.

## Methods

This retrospective study was carried out at the emergency centre of a referral hospital. This hospital provides burn care services for over a thousand people and is one of the biggest referral burn centers located in the urban area Malang, East Java Province, Indonesia. Malang City is a city located in East Java Province, Indonesia, the second largest city in East Java after Surabaya, and the 12th largest city in Indonesia. This city was founded during the Kanjuruhan Kingdom era and is situated on a plateau covering an area of 145.28 km^2^ located in the middle of Malang Regency. The study was approved by the ethical committee of Saiful Anwar General Hospital. Medical records of burn patients admitted to the emergency centre from April 2017 to April 2018 which fulfilled inclusion criteria were collected.

The criteria of inclusion were complete folders of patients with age older than 17 years old, gender, and social condition. Data regarding preexisting medical conditions, regardless of the relation to the burn trauma without chemical and ocular burns, were collected. Demographic information, TBSA, overall TBSA, inhalation injury, and in hospital mortality were recorded. Researchers who collected the data and/or computed the burn scores were trained and experienced using different burn scoring systems both in academic and clinical practices.

This study used four of the most common scoring systems to predict the risk of death: the Ryan Model, the Revised Baux Score, BOBI, and ABSI. The Ryan Model [15] uses 3 parameters (age, total burn surface area and inhalation injury) to assess the risk of mortality. An increasing number of risk factors (0–3) are associated with an increasing mortality rate. When there is no risk factor, mortality rate is 0.3%. One risk factor corresponds to 3%, two risk factors correspond to 33%, and 3 risk factors give a probability of death of 90%. The Revised Baux Score [16] adds the number 17 to the sum of the patient's age and TBSA. Risk of mortality goes from 0 to 100%. Introducing a new nomogram to calculate the Revised Baux Score has simplified its usage. BOBI [17] uses age, TBSA and presence of inhalation injury as three risk factors. Age is divided into four groups (0–3 points) and TBSA into 5 groups (0–4 points). Presence of inhalation injury takes 3 points. Based on total score (0–10 points), predicted mortality ranges between 0.1% and 99%. ABSI [18] uses five variables to predict prognosis: increased TBSA (1–10 points), increased age (1–5 points), female gender (1 point), presence of inhalation injury (1 point), and presence of full-thickness burns (1 point). The sum of these values ranges from two to 18 points, and survival probability percentage decreases as the score increases (≤10% and ≥99%).

Comparison of demographic and burn characteristics between survivors and deceased patients was performed. Comparison of quantitative continuous variables was carried out using the sample *t*-test, and categorical variables were compared using the chi square test. Data are expressed as number (%) or mean ± standard deviation (SD). Hosmer and Lemeshow Chi-square statistical test were used to calculate the prediction models' accuracy and goodness-of-fit. The region under the curve (AUC) was used to identify the model and was much more accurate in the distinction between survivors (false positives) and deceased (true positive). An area >0.9 indicated high accuracy, 0.7–0.9 moderate accuracy, 0.5–0.7 low accuracy, and 0.5 indicated discrimination of chance [19]. Statistical significance was defined as a *p* value<0.05. Statistical analysis was carried out using the Statistical Package for Social Sciences (SPSS 22, SPSS Inc., Chicago, US).

## Results

From April 2017 to April 2018, 111 patients were admitted to the emergency centre. Data included for analysis were obtained from 72 of the 111 burns patients (64.9%) whereas other data were excluded from 39 (35.1%). The excluded data were 11 incomplete medical records, 13 related ocular burns, and 15 children patients. Of all 72 patients, a total of 18 deaths were recorded, with an overall mortality of 25%.

Characteristics of patients are presented in Table 1. The patients were 44 males (61.1%) and 28 females (38.9%), with an average age of 41 years (range 18–82 years). Survivors were younger (39 ± 14 vs.45 ± 21.06) and lower TBSA (14 ± 14.04 vs. 52 ± 28) than those deceased (*p* < 0.05). About 4.2% of survivors had full-thickness burns, which was significantly higher than those deceased (p < 0.05).

**Table 1:** Characteristics burn patients (*n* = 72).

Characteristics	All (n = 72)	Survivor (n = 54)	Deceased (n = 18)	p-Value
Age (year) (mean ± SD)	40.79 ± 16.30	39 ± 14	45 ± 21.06	0.0001
Gender, n (%)				0.027
Male	44 (61.1)	32 (59.2)	12 (66.7)	
Female	28 (38.9)	22 (40.7)	6 (33.3)	
TBSA (%) (mean ± SD)	23.59 ± 24.84	14 ± 14.04	52 ± 28	0.0001
Inhalation injury				
Yes	1 (1.4)	1 (1.9)	0 (0)	0.0001
No	71 (98.6)	53 (98.1)	18 (100)	
Full thickness burn				
Yes	3 (4.2)	3 (4.2)	0 (0)	0.0001
No	69 (95.8)	51 (94.4)	18 (100)	

TBSA, total body surface area.

**Fig. 1:**
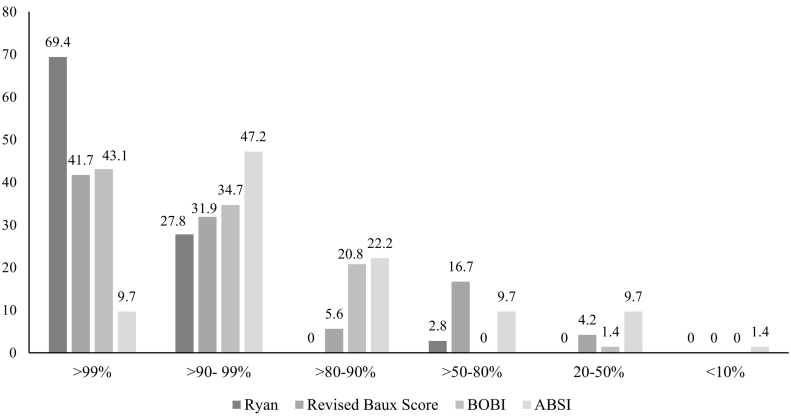
Distribution frequency of probability of survival as measured by different Burn Scoring Systems (*n* = 72).

In a comparison between survivors and deceased victims (Table 1), the survivors (*n* = 54) had a significantly lower mean age, %TBSA, and number of inhalation injuries (*p* < 0.001). The probabilities of survival (>99%) predicted by the Ryan model, Revised Baux Score, BOBI, and ABSI were respectively 69.4%, 41.7%, 43.1%, 9.7% (Fig. 1). The best prediction of mortality percentage was estimated by the ABSI model. Sensitivity was 81.6, specificity was 92.5, accuracy was 87.3 and under the Receiver Operator Characteristics curve (AUC) was 0.93 (SE = 0.03) (Table 2).

**Table 2:** Sensitivity, specificity, accuracy, Hosmer-Lemeshow goodness of fit (H-L), and area under the Receiver Operator Characteristics curve (AUC) among different burn scoring systems.

Score	Sensitivity	Specificity	Accuracy	H-L (p)	AUC ± SE
Ryan	78.2	97.8	75.6	6.78 (0.051)	0.80 ± 0.06
BRAUX Revised	77.6	93.6	85.2	5.01 (0.067)	0.89 ± 0.03
BOBI	73.1	91.8	84.1	9.89 (0.451)	0.90 ± 0.04
ABSI	81.6	92.5	87.3	6.79 (0.537)	0.93 ± 0.03

BOBI, Belgian Outcome in Burn Injury; ABSI, Abbreviated Burn Severity Index.

## Discussion

This study applied four burn injury scoring systems and their accuracy to predict to in-hospital mortality. Our findings suggested that abbreviated burn severity index (ABSI) demonstrated high accuracy compared to Ryan, Revised Baux Score, BOBI. This finding supports previous studies which showed good performance of ABSI [[20], [21], [22]]. The ABSI scoring system remains an accurate and valuable method for determining which is in line with other Swiss Burn Center results [22]. Although Ryan, Revised Baux Score, BOBI also showed acceptable accuracy, in clinical practice, ABSI seems to be more easily implemented.

This study also indicated weaker BOBI score outcomes, especially in comparison to other systems in our study [20]. Our results contrast with those of Douglas et al. [23] who identified that the BOBI score was more accurate than the revised Baux score on a cohort of 48 patients. Discrepancies with earlier findings in this result could be due to the differences in demographic and quality of care in each area. It is therefore very crucial to analyse the internal or external validity of the prediction models before they are used in a new population or case mix [22].

Compared with survivors, deceased patients had a significantly higher mean age, %TBSA, and number of inhalation injuries (*p* < 0.001). These three important factors are the foundation of nearly all specific burn prediction models. Consequently, the importance of these prognostic factors in burn injuries was confirmed by our study. The AUC for the five specific outcome models demonstrated moderate accuracy at distinguishing between survivors and non-survivors, so the majority of burn injured patients who died were expected to die. In this study, true mortality was 18 (25%). Healing in IIA burn degree occurs spontaneously within 10–14 days without circulation [8]. While healing in IIB burn degree occurs in more than one month. Most patients with <25% burns can be managed without treatment in the critical care room. For the management of burns, the majority of patients with burns require long-term scar management. This is related to the extent of burns experienced by patients: a higher degree of severity of burns extends the duration of treatment in the hospital, and the majority of patients with burns are not treated intensively [11].

There are some limitations to the study. First, this research was a retrospective study conducted in a central location and thus has limitations on controlling other confounding factors because it relies on existing data. However, since there was a higher mortality of burn cases, a retrospective study may have benefits for its accessibility, easiness, and timeless compared to a prospective study. For mortality research, the number of patients included with the study (*n* = 72) may well be considered as low. Relevant survival differences (type I error, or alpha error) can then be unreported. Although this cohort consisted of consecutive admissions to an intensive care unit, according to rigorous entrance requirements, other total scores have been reported in patients who were seriously ill enough just to rely solely on critical care therapy. Additionally, our normal protocol places special focus on airway obstruction lesions and involves a low tracheal intubation threshold accompanied by early endotracheal intubation when clinically necessary. The subsequent rise in inhalation injury treatment certainly affected the individual burn scores, and their accuracy declined.

Our analysis shows that specific predictive scores in this sample of intensive care units supported the excellent performance of the ABSI ratings. Ryan, revised BAUX, and BOBI scores were also good, but it is important to note that their performance is closely linked to the inhalation injury concept. It varies widely from one burn center to another and across various countries. Eventually, the ABSI score has proven almost as accurate and sensitive as the revised Baux, Ryan and BOBI. This proves the importance of critical care in the survival of those patients most severely burned. In addition, this finding could be applied in other countries, particularly low-income countries with similar conditions as Indonesia that can use ABSI in a clinical setting. Still, it should also be taken into consideration that its performance in severely burned patients is declining.

## Dissemination of results

The results of this study were discussed informally with the medical professionals at the study location.

## Authors' contribution

Authors contributed as follow to the conception or design of the work; the acquisition, analysis, or interpretation of data for the work; and drafting the work or revising it critically for important intellectual content: RH contributed 50%; EP, IW, and IDP contributed 16.6% each. All authors approved the version to be published and agreed to be accountable for all aspects of the work.

## Declaration of competing interest

All authors declare no conflict of interest.
